# Higher incidence of hypotension episodes in women during the sub-acute phase of ST elevation myocardial infarction and relationship to covariates

**DOI:** 10.1371/journal.pone.0173699

**Published:** 2017-03-09

**Authors:** Petr Kala, Tomas Novotny, Irena Andrsova, Klara Benesova, Maria Holicka, Jiri Jarkovsky, Katerina Hnatkova, Lumir Koc, Monika Mikolaskova, Tereza Novakova, Tomas Ondrus, Lenka Privarova, Jindrich Spinar, Marek Malik

**Affiliations:** 1 Department of Internal Medicine and Cardiology, University Hospital Brno and Faculty of Medicine of Masaryk University, Brno, Czech Republic; 2 Institute of Biostatistics and Analyses, Faculty of Medicine of Masaryk University, Brno, Czech Republic; 3 National Heart and Lung Institute, Imperial College, London, United Kingdom; University of Bologna, ITALY

## Abstract

**Objective:**

The introduction of primary percutaneous coronary intervention (PPCI) has modified the profile of ST elevation myocardial infarction (STEMI) patients. Occurrence and prognostic significance of hypotension episodes are not known in PPCI treated STEMI patients. It is also not known whether and/or how the hypotension episodes correlate with the degree of myocardial damage and whether there are any sex differences.

**Methods:**

Data of 293 consecutive STEMI patients (189 males) treated by PPCI and without cardiogenic shock were analyzed. Blood pressure was measured noninvasively. A hypotensive episode was defined as a systolic blood pressure below 90 mmHg over a period of at least 30 minutes.

**Results:**

A hypotensive episode was observed in 92 patients (31.4%). Female sex was the strongest independent predictor of hypotension episodes (p < 0.0001), while there was no relationship to electrocardiographic STEMI localization. Hypotensive patients had significantly higher levels of troponin T and brain natriuretic peptide; hypotensive episodes were particularly frequent in women with increased troponin T. Treatment with angiotensin-converting enzyme inhibitor (ACEI), angiotensin receptor blocker (ARB) and betablockers was less frequent in hypotensive patients. After a mean 20-month follow-up, all-cause mortality did not differ between hypotensive patients and others. However, mortality in hypotensive patients who did not tolerate ACEI/ARB therapy was significantly higher compared to other hypotensive patients (p = 0.016).

**Conclusion:**

Hypotension episodes are not uncommon in the sub-acute phase of contemporarily treated STEMI patients with a striking difference between sexes—female sex was the strongest independent predictor of hypotension episodes. Hypotensive episodes may lead to a delay in pharmacotherapy which influences prognosis. Higher incidence of hypotension in women could at least partially explain the sex-related differences in the use of cardiovascular pharmacotherapy which was repeatedly observed in various studies.

## Introduction

Arterial hypotension is an important complication in ST elevation myocardial infarction (STEMI) [1] The majority of existing data and experience with hypotension in STEMI patients were mainly obtained prior to the introduction of primary percutaneous coronary intervention (PPCI). In particular, hypotension has long been recognized as a complication of streptokinase administration to STEMI patients [2]. Compared to the thrombolytic treatment, PPCI improved the prognosis of STEMI patients substantially [3,4]. Consequently, PPCI is presently the standard treatment of choice in all acute STEMI patients. This change of clinical practice has modified the sub-acute profile of STEMI patients. Among others, occurrence and prognostic significance of hypotension episodes are not known in PPCI treated STEMI patients. It is also not known whether and/or how the hypotension episodes correlate with the degree of myocardial damage and whether there are any sex differences. Having this in mind, we monitored the incidence of hypotension episodes in a consecutive population of STEMI patients hospitalized between December 2012 and April 2015.

## Methods

### Patients

From total of 300 patients enrolled in the bilateral Holter-ECG project, data of 293 consecutive STEMI patients (189 males) were analyzed (7 patients were excluded from the analysis because of very short period of blood pressure monitoring). All the patients were referred to our coronary catheterization laboratory with the diagnosis of acute STEMI fulfilling the criteria for PPCI according to guidelines [5,6]. Localization of infarction was categorized as anterior (leads V_1_-V_4_), inferior (II,III,aVF), lateral (I,aVL and/or V_5_-V_6_), and septal (V_1_-V_2_). Cases of non-obstructive coronary artery disease (i.e. Takotsubo cardiomyopathy) were not included in the study. Patients unable (in cardiogenic shock and/or unconscious on hospital admission) and those unwilling to sign an informed consent were excluded. The time to reperfusion was defined as the interval between symptom onset and the wire passage in the culprit artery. Time of acute MI onset was defined as that of first chest pain symptoms. After the PPCI procedure, the patients were hospitalized in the coronary care unit for at least 72 hours. An attempt of an initiation of angiotensin-converting enzyme inhibitor (ACEI) or angiotensin receptor blocker (ARB) and/or betablocker therapy was done immediately after transfer of a patient to coronary care unit (in the absence of contraindications, namely hypersensitivity, hypotension, history of angioneurotic oedema for ACEI/ARB, and hypersensitivity, higher grade atrioventricular block, severe sinus bradycardia, hypotension, acute heart failure, asthma bronchiale, obstructive pulmonary disease for BB, respectively). The project was approved by the Ethics committee of University Hospital Brno, all patients signed an informed consent.

### Clinical measurements

Noninvasive blood pressure monitoring was performed automatically (Dash 4000 Patient Monitor, GE Medical Systems, Milwaukee, WI, U.S.A.) at least every 30 minutes. Heart rates were measured at the same time. A hypotensive episode was defined as a systolic blood pressure below 90 mmHg over a period of at least 30 minutes when criteria for cardiogenic shock were not fulfilled.5 If a patient suffered from repeated hypotension episodes, only the timing of the first episode was considered. The decision of therapeutic intervention was based on individual opinion of a particular physician on duty.

Body mass index (BMI) was calculated as BMI = W/H^2^; lean body mass [7] (LBM) was calculated as LBM = 0.29569*W + 41.813*H—43.2933 for females and LBM = 0.3281*W + 33.929*H—29.5336 for males, respectively; and body surface area (BSA) was calculated as the average of the Du Bois and Du Bois formula and of the Gehan and George formula [8], that is BSA = 0.101236*W^0.425^*H^0.725^ + 0.082216*W^0.51456^*H^0.42246^, where W is body weight in kilograms and H is body height in metres.

Troponin T levels were measured 24 hours after the chest pain onset (Troponin T high sensitivity assay, Roche Diagnostics, Basel, Switzerland). Brain natriuretic peptide (BNP) levels were assessed in the morning of the second hospitalization day (Architect BNP assay, Abbott Laboratories, Chicago, IL, USA). Left ventricular ejection fraction (LVEF) was measured by echocardiography before discharge. Body mass index (BMI) was calculated as the body mass divided by the square of the body height. Data on all-cause mortality were retrieved from the Nation-wide health insurance registry.

### Statistics

Absolute and relative frequencies were obtained for categorical variables; mean±standard deviation and median with minimum to maximum range were used to characterize continuous variables. Differences between patients with and without hypotension were tested using Fisher’s exact test for categorical variables and non-parametric Mann-Whitney U test for continuous variables. In patients with hypotension episodes, averages of two heart rate measurements during the episode were compared with the averages of two heart rate measurements before the episode using Wilcoxon paired test. Spearman correlation coefficients were calculated between troponin T levels and BSA. To associate hypotension episodes with age, BMI, LBM, BSA, troponin T levels, and BNP levels, the incidence of the episodes was compared between patients above and below median of the respective variables. The tests were also repeated separately for female and male patients with median cut-offs obtained separately in females and males. Incidence of hypotension episodes and all-cause mortality were evaluated by Kaplan-Meier survival event-free curves and compared by the log-rank test. Multivariable backwards stepwise regression analysis of prediction of hypotension episodes was performed in two modes: using original values of numerical variables and dichotomizing non-binary variables at population medians. Statistical analyses were performed using IBM SPSS Statistics for Windows, Version 22.0.0.1, and in Statistica package, Version 6.1. P level <0.05 was considered statistically significant.

## Results

Table 1 shows clinical characteristics of the population and the principal results. The PPCI procedure of infarction artery was successful in all investigated individuals. No patient developed cardiogenic shock during hospitalization.

**Table 1:** Comparison of patients with and without hypotension.

Parameter		Total (N = 293)	No hypotension (N = 201)	Hypotension(N = 92)	P
Sex	Men	189 (64.5%)	148 (73.6%)	41 (44.6%)	**< 0.001**
	Women	104 (35.5%)	53 (26.4%)	51 (55.4%)	
Age (years)	(N = 293)	62.8 ± 12.0	61.6 ± 11.7	65.5 ± 12.1	**0.015**
		62.1 (30.7; 89.5)	61.2 (30.7; 88.4)	65.6 (35.5; 89.5)	
BMI (kg/m^2^)	(N = 293)	28.6 ± 4.6	29.0 ± 4.3	27.6 ± 5.1	**0.002**
		28.1 (19.5; 51.0)	28.6 (21.1; 48.4)	27.3 (19.5; 51.0)	
LBM (kg)	(N = 293)	55.21 ± 8.47	57.16 ± 7.91	45.42 ± 4.92	**< 0.001**
		55.72 (36.65; 76.51)	57.36 (36.65; 76.51)	45.19 (37.78; 61.33)	
BSA (m^2^)	(N = 293)	1.99 ± 0.22	2.04 ± 0.21	1.78 ± 0.16	**< 0.001**
		1.98 (1.5; 2.58)	2.01 (1.52; 2.58)	1.76 (1.5; 2.34)	
Previous MI		27 (9.2%)	17 (8.5%)	10 (10.9%)	0.519
Previous PCI/CABG		27 (9.2%)	19 (9.5%)	8 (8.7%)	0.999
Hypertension		173 (59.0%)	123 (61.2%)	50 (54.3%)	0.306
Dyslipidemia		163 (55.6%)	123 (61.2%)	40 (43.5%)	**0.005**
Diabetes		64 (21.8%)	47 (23.4%)	17 (18.5%)	0.366
AMI localization*	Anterior	127 (43.3%)	88 (43.8%)	39 (42.4%)	0.899
	Inferior	144 (49.1%)	95 (47.3%)	49 (53.3%)	0.379
	Lateral	48 (16.4%)	33 (16.4%)	15 (16.3%)	0.999
	Septal	6 (2.0%)	6 (3.0%)	0 (0.0%)	0.182
	Posterior	11 (3.8%)	8 (4.0%)	3 (3.3%)	0.999
Troponin T max. (ng/ml)	(N = 290)	3.4 ± 3.3	2.9 ± 3.0	4.4 ± 3.6	**< 0.001**
		2.4 (0.0; 20.9)	1.8 (0.0; 20.9)	3.5 (0.0; 18.1)	
BNP (pg/ml)	(N = 275)	412.6 ± 412.8	358.5 ± 396.9	524.0 ± 424.5	**< 0.001**
		283.9 (27.5; 3 129.0)	233.3 (30.8; 3 129.0)	388.0 (27.5; 2 313.0)	
Time to reperfusion therapy (in hours)	(N = 293)	6.9 ± 4.2	5.0 ± 4.5	4.9 ± 3.4	0.261
		3.4 (1.0; 24.6)	3.3 (1.1; 24.6)	3.7 (1.0; 23.0)	
Hypotension therapy	None	-	-	63 (68.5%)	-
	Volumotherapy	-	-	10 (10.9%)	-
	Vasocative agents	-	-	6 (6.5%)	-
	Both	-	-	13 (14.1%)	-
LVEF (%) at discharge	(N = 291)	52.1 ± 10.0	53.3 ± 10.1	49.7 ± 9.5	**0.004**
		55.0 (25.0; 79.0)	55.0 (25.0; 79.0)	50.0 (30.0; 70.0)	
Length of follow-up (in months)	(N = 293)	20.2 ± 8.1	19.7 ± 8.2	21.1 ± 7.7	0.194
		18.9 (0.7; 34.6)	18.7 (0.7; 34.6)	19.8 (0.7; 34.5)	

Categorical variables are described by absolute and relative frequencies; mean (± SD) and median (min; max) are shown for continuous variables. P-value of Fisher’s exact test and P-value of Mann-Whitney U test are shown for categorical and continuous variables, respectively.

* Multiple localizations are possible.

AMI = acute myocardial infarction, BMI = body mass index, BNP = brain natriuretic peptide, BSA = body surface area, CABG = coronary artery bypass surgery, LBM = lean body mass, LVEF = left ventricular ejection fraction, PCI = percutaneous coronary intervention.

**Fig 1:**
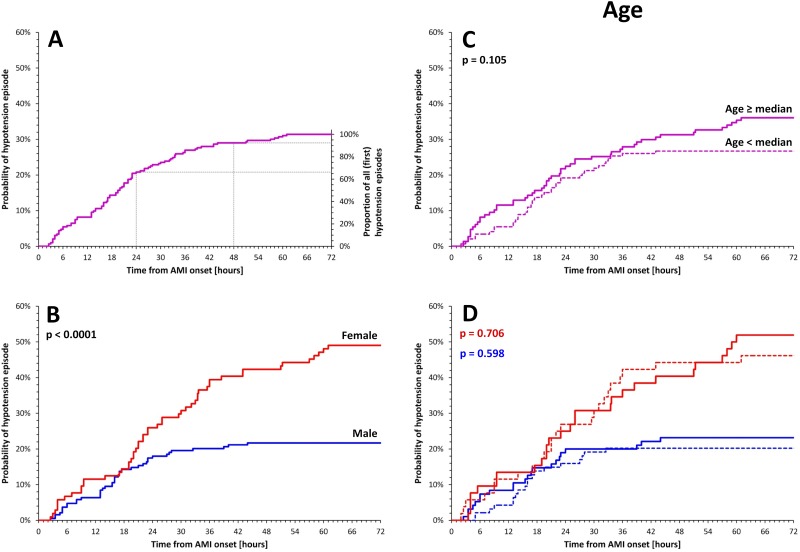
Panel A—probability of hypotension episodes in 92 patients. Panel B—differences between females (red line) and males (blue line). Panel C—the differences in patients above (continuous line) and below (dotted line) median age in the complete population. Panel D—the age-related analysis shown separately in females (red lines) and males (blue lines). Continuous and dashed lines show patients above and below age median, respectively. AMI—acute myocardial infarction.

During the 72 hours a hypotension episode was observed in 92 patients (31.4%). In 17 (18.5%) of them, hypotensive values were present immediately after the transfer from catheterization laboratory. Approximately two thirds of the episodes occurred during the first 24 hours compared to fewer than 10% during the third hospitalization day (Fig 1A).

### Univariable correlates

Hypotension episodes were significantly more frequent in females (Fig 1B). Simple statistical comparison (Table 1) suggested that hypotension was also more frequent in elderly patients. However, (Fig 1C) only a non-significant trend towards more frequent episodes was observed in patients aged above and below 62.1 years (complete population median). Moreover, female patients were significantly older than males (68.0±10.4 vs. 59.9±11.8 years, p < 0.0001) and when distinguishing women and men above and below median age of 67.5 and 58.8 years, respectively, no trend towards more frequent hypotension episodes in older patients was visible (Fig 1D).

Occurrence of hypotension was not related to electrocardiographic STEMI localization. There was no relationship to the time to reperfusion.

Heart rates were marginally non-significantly lower within the hypotension episodes in comparison to the measurements before the episodes (heart rate reduction by 0.88±4.18 vs. 0.30±5.53 beats per minute in males and females, respectively; p = NS for comparison between sexes).

**Fig 2:**
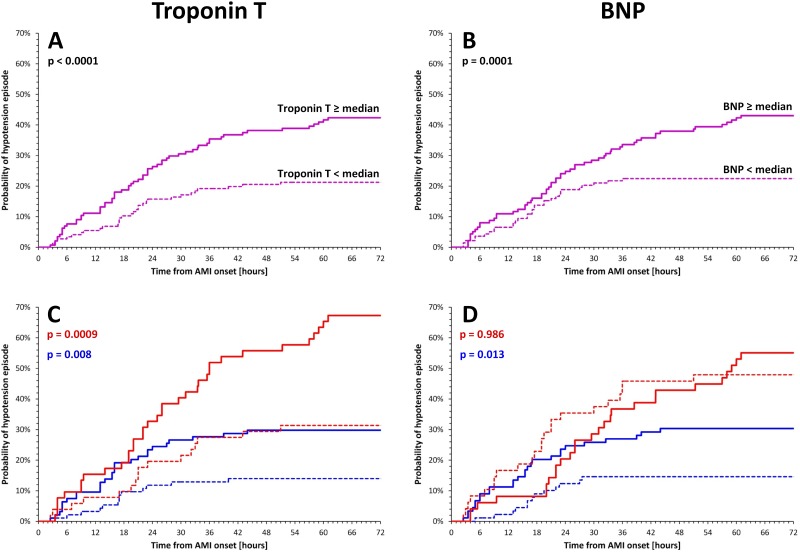
Panel A—probability of hypotension episodes in patients above (continuous line) and below (dashed line) troponin T median of the complete population. Panel B—the differences according to BNP values above (continuous line) and below (dashed line) median of the complete population. Panel C—troponin T-related analysis shown separately in females and males. Panel D—BNP-related analysis shown separately in females and males. In panels C and D, red and blue lines show female and male subgroups, respectively, the continuous and dashed lines show patients above and below sex-specific median values, respectively. BNP—brain natriuretic peptide, AMI—acute myocardial infarction.

Hypotensive patients had significantly higher levels of troponin T (population median of 2.39 ng/ml, Fig 2A) and BNP (population median of 284 pg/ml, Fig 2B), and lower LVEF. Women had troponin T levels similar to those in men (3.33±3.17 vs. 3.41±3.43 ng/ml, p = NS); after adjusting troponin T levels linearly for BSA, only a non-significant trend towards higher values in women was observed (1.95±2.22 vs. 1.67±1.58 ng/ml/m^2^, p = NS). Also, troponin T levels did not significantly correlate with BSA or LBM (in the total population as well as in sex-specific subgroups). Nevertheless, when distinguishing women and men above and below the sex-specific medians of troponin T (2.38 and 2.41 ng/ml, in women and men, respectively) the separation of the hypotension incidence became more apparent (Fig 2C). In particular, there was no difference in hypotension incidence between women with lower and men with higher troponin T levels. Such a distinction was not observed for BNP levels that were much higher in women than in men (550±392 vs. 338±405 pg/ml, p<0.0001). While there was a difference in the incidence of hypotension episodes in men with BNP levels above and below sex-specific BNP median of 212 pg/ml, the incidence did not differ between women with BNP levels above and below 510 pg/ml (median in women) (Fig 2D).

Hypotensive individuals had also a lower BMI. However, separating patients with lower and higher BMI did not show any significant difference in hypotension incidence. The same was true for the separate sex-groups; women and men did not differ in BMI (28.05±4.97 vs. 28.71±4.37 kg/m^2^, p = NS). Of the medical history data, the only difference between patients with and without hypotension episodes was a less common dyslipidemia in hypotensive patients.

**Fig 3:**
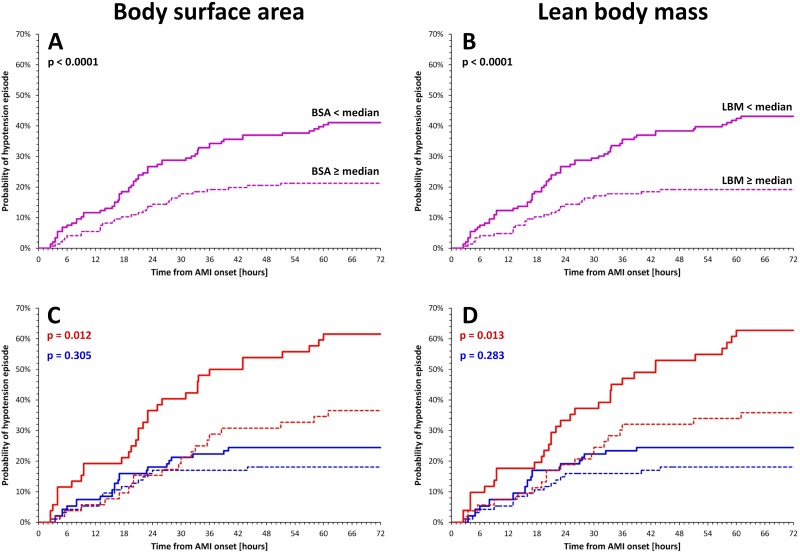
Panel A—probability of hypotension episodes in patients below (continuous line) and above (dashed line) BSA median of the complete population. Panel B—the differences according to LBM values below (continuous line) and above (dashed line) median of the complete population. Panel C—BSA-related analysis shown separately in females and males. Panel D—LBM-related analysis shown separately in females and males. In panels C and D, red and blue lines show female and male subgroups, respectively, the continuous and dashed lines show patients below and above sex-specific median values, respectively. BSA—body surface area, LBM—lean body mass, AMI—acute myocardial infarction.

Since compared to men, women had significantly lower LBM (47.07±5.65 vs. 59.52±6.30 kg p<0.001) and smaller BSA (1.83±0.18 vs. 2.08±0.19 m^2^, p<0.001) it was not surprising that patients with LBM below population median and with BSA below population had more frequent hypotension episodes (Fig 3A and 3B). However, when repeating these analyses for separate sex groups, significantly higher incidence of hypotension episodes was also observed in women with LBM below sex-specific median of 47.2 kg and in women with BSA below sex-specific median of 1.83 m^2^. In men, distinctions between patients with LMB and BSA below sex-specific median showed only non-significant trends towards higher hypotension incidence among those with lower LBM or lower BSA (Fig 3C and 3D).

### Multivariable analyses

The multivariate regression analysis considered sex, age, BMI, LBM, BSA, hypertension, troponin T, BNP, and LVEF values as predictors of hypotension episodes. Dichotomized analysis used the following population medians for age, BMI, LBM, BSA, troponin T, BNP, and LVEF: 62.1 years, 28.1 kg/m^2^, 2.39 ng/ml, 283.9 pg/ml, and 55%, respectively.

With both analysis models, only sex and troponin T levels were consistently significant predictors of hypotension (p < 0.0001). In both models, sex differences were stronger predictor (F statistics of 27.9 and 28.3 for continuous and dichotomized models, respectively) than the troponin T levels (F of 14.9 and 19.2, respectively).

### Treatment effects

In 29 (31.5%) patients with hypotension, the event required therapy by volume expansion and/or vasoactive drugs (Table 1). Of all 92 hypotension patients, 28 (30.4%) suffered from repeated episodes.

**Fig 4:**
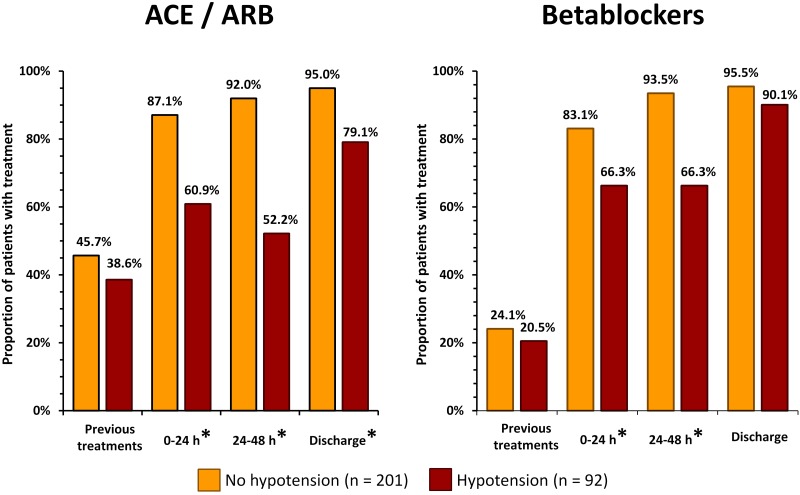
Proportions of patients treated with ACEI/ARB and betablockers are shown before the admission due to acute STEMI (previous treatment), during first and second day of hospitalization, and at discharge separately in women (top panels) and men (bottom panels). Statistically significant differences between patients with and without hypotension are marked with asterisk. STEMI—ST elevation myocardial infarction, ACEI—angiotensin-converting enzyme inhibitor, ARB—angiotensin receptor blocker.

The proportion of ACEI/ARB and betablocker therapy was lower in hypotensive individuals during hospitalization. At discharge the difference remained significant in ACEI/ARB. A trend for lower use of ACEI/ARB in women at discharge was non-significant (86.4% vs 92.1%, p = NS). However, when distinguishing hypotensive and non-hypotensive patients, the proportions of ACEI/ARB and betablocker therapy did not differ between women and men. However, prior ACEI/ARB treatment was more frequent in women compared to men (p = 0.036 and p = 0.048 for patients without and with hypotension, respectively). Consequently, the difference in discharge versus prior ACEI/ARB treatment in women with hypotension (30%) was much lower compared to men with hypotension (54.2%) (Fig 4).

### Follow-up

**Fig 5:**
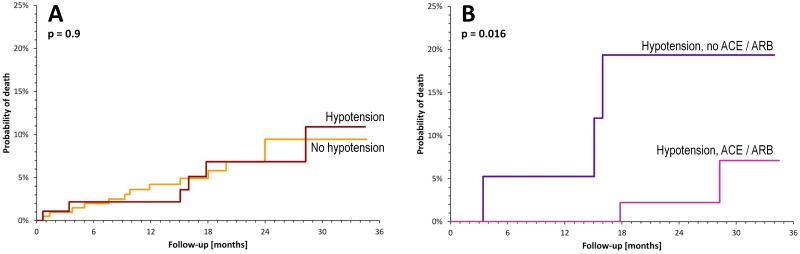
Kaplan-Meier analysis of probability of death in study patients. Panel A—comparison of patients without (yellow line) and with hypotension episodes (brown line). Panel B—a comparison of hypotension patients with (violet line) and without ACEI/ARB treatment (blue line) at discharge. ACEI—angiotensin-converting enzyme inhibitor, ARB—angiotensin receptor blocker.

After an average 20-month follow-up, the all-cause mortality was similar in patients with and without hypotension episodes (Fig 5A). Nevertheless mortality of hypotensive patients who did not tolerate ACEI/ARB was significantly higher compared to other hypotensive patients (Fig 5B).

## Discussion

STEMI patients with impaired hemodynamics are at increased risk of life-threatening complications and their prognosis is uncertain [5,6]. Although hypotension is a well-recognized risk factor in the early stage of STEMI, its significance in the subacute phase is much less known [1].

In our population of PPCI treated STEMI patients, hypotension episodes were not uncommon and occurred in approximately one third of all patients. Increased levels of troponin T and BNP suggested higher amount of damaged myocardium[9], as further supported by lower LVEF at discharge.

The female sex was found to be the strongest independent predictor of hypotension episodes. The reasons for this striking sex-related difference are not immediately obvious. It has been hypothesized that the stiffer and thicker ventricles in women tolerate less the volume shifts often occurring during early STEMI phases [10]. While women were substantial older than men, age had no influence on the incidence of the episodes in sex-separated sub-groups.

Troponin levels after STEMI correlate with myocardial performance by echocardiography [11]. Not surprisingly, increased troponin levels were also independently predicting hypotension episodes. In healthy population or stable coronary disease, women have generally lower troponin T levels [12,13]. There are no such data in PPCI treated STEMI patients. In our study, troponin T levels were similar in both sexes. While women have smaller left ventricular mass similar troponin T levels may suggest greater myocardial damage compared to men, as supported by recent data [14]. These facts could explain our finding that women with higher troponin T levels had markedly higher incidence of hypotension episodes, although we observed only non-significantly increased troponin T levels adjusted for BSA in women. Levels of BNP in women were significantly higher but the incidence of hypotension was not different with values above and below median. On the contrary this difference was apparent in men. There are limited data on sex-specific BNP differences during acute coronary syndromes. In a large registry, women had higher BNP at admission due to heart failure than men, when stratified by LVEF [15]. These observations suggest that women develop more pronounced heart failure (measured by BNP levels) compared to men with similar amount of myocardial damage (measured by troponin T levels) and these women are also more prone to hypotension. The reason for the observation of increased incidence of hypotension episodes is women with lower LBM and smaller BSA is not immediately obvious. It might be related to the lower volume of circulating blood and pool of body liquids that might offer less buffering for mechanisms compensating blood pressure decreases. Since no differences were observed among patients stratified according to BMI, the observation is probably unrelated to the known increased cardiovascular risk in leaner patients [16].

Apart from the observations of greater myocardial damage, there are no clear mechanistic explanations for these findings as there was no difference in time to reperfusion and no relationship to MI localization although it would be plausible to expect higher occurrence of hypotension particularly in anterior MI, traditionally indicating increased infarction size. It could be speculated that such paradigms are changing with PPCI therapy. Often assessed comorbidities of hypertension, diabetes, previous MI, previous PCI, and coronary artery bypass surgery, did not differ between hypotensive and normotensive patients (Table 1). The observation of less frequent dyslipidemia among hypotensive patients was likely only a collateral chance finding.

Numerous studies have demonstrated a benefit of early initiation of betablocker and ACEI/ARB therapy in STEMI patients [17,18]. In our study, episodes of hypotension prevented early administration of ACEI, ARB and betablockers in a substantial sub-group of patients. With decreasing occurrence of hypotensive events during the first 72 hours after MI, the proportion of treated patients was increasing. At discharge, more than 90% of hypotensive as well as normotensive patients were treated by betablockers. However, the proportions of ACEI/ARB treated patients were different. In the hypotensive group, a proportion of ACEI/ARB treatment reached 79.1% at discharge. Although this percentage was not particularly low, it was significantly lower compared to the normotensive group (95%). Multiple studies have shown that women with acute coronary syndrome are less likely to be treated with guideline-directed medical therapies [19,20]. In our study, a simple statistical assessment showed a similar trend for lower use of ACEI/ARB treatment in all women at discharge. Higher incidence of hypotension in women could at least partially explain the previously observed sex-related differences in the use of cardiovascular pharmacotherapy.

The all-cause mortality was low. After a mean 20-months follow up, it did not differ between hypotensive and normotensive patients. Nevertheless in the hypotensive patients without ACEI/ARB, the mortality was significantly increased despite relatively short follow-up. These findings emphasize the necessity for efforts to initiate ACEI/ARB therapy even in hypotensive patients.

### Limitations

This is a single center experience, the number of investigated patients was relatively small preventing many sub-group analyses. Blood pressure was not measured continuously. No distinction of how deep systolic blood pressure decreased below 90mmHg was considered but none of the patients suffered from a newly developed cardiogenic shock. Durations of the hypotensive episodes were not considered. The averaged follow-up was only 20 month. The incidence of hypotension was measured from MI onset although the regular blood pressure measurement started only after hospitalization. Multivariable regression models do not consider the time between index MI and the hypotension episodes. Nevertheless, we also calculated proportional hazard Cox regression models and obtained the very same results.

## Conclusion

In spite of these limitations, the study permits to conclude that hypotension episodes are not uncommon in the sub-acute phase of PPCI treated STEMI patients. Striking gender specific differences in hypotension incidence and its relationship to troponin levels were observed. Episodes of hypotension may delay the start of pharmacotherapy. Patients in whom hypotension prevented administration of ACEI/ARB had a significantly poorer prognosis. Higher incidence of hypotension in women could at least partially explain the sex-related differences in the use of cardiovascular pharmacotherapy which was repeatedly observed in various studies.
